# Improved heat coefficients for joint-space metabolic energy expenditure model during level, uphill, and downhill walking

**DOI:** 10.1371/journal.pone.0267120

**Published:** 2022-04-14

**Authors:** Jazmin Cruz, James Yang

**Affiliations:** Department of Mechanical Engineering, Human-Centric Design Research Lab, Texas Tech University, Lubbock, TX, United States of America; University degli Studi di Milano, ITALY

## Abstract

A previously developed joint-space metabolic energy expenditure (MEE) model includes subject-specific parameters and was validated using level walking gait data. In this work, we determine how well this joint-space model performs during various walking grades (-8%, 0%, and 8%) at 0.8 m·s ⁻^1^ and 1.3 m·s ⁻^1^ using published gait data in the literature. In response to those results, we formulate an optimization problem and solve it through the particle swam method plus *fmincon* function in MATLAB to identify a new optimal weighting parameter set for each grade that produces more accurate predicted MEE and we compare our new findings with seven other MEE models in the literature. The current study matched the measured MEE the best with the lowest RMSE values for level (0.45 J·kg ⁻^1^·m ⁻^1^) and downhill (0.82 J·kg ⁻^1^·m ⁻^1^) walking and the third lowest RMSE value for uphill (1.56 J·kg ⁻^1^·m ⁻^1^) walking, where another MEE model, Looney et al., had the lowest RMSE for uphill (1.27 J·kg ⁻^1^·m ⁻^1^) walking. Bland-Altman plots and three independent-samples t-tests show that there was no statistical significant difference between experimentally measured MEE and estimated MEE during the three walking conditions, meaning that the three new optimal weighting parameter sets can be used with 6 degree of freedom (DOF) lower extremity motion data to better estimate whole body MEE in those scenarios. We believe that this work is a step towards identifying a single robust parameter set that allows for accurate estimation of MEE during any task, with the potential to mitigate a limitation of indirect calorimetry requiring lengthy steady state motion.

## 1. Introduction

The ability to estimate metabolic energy expenditure (MEE) is desirable for many reasons. This measurement could inform physical laborers on when to stop a particular task, especially for those in the military. Gaining insight on how much energy a task requires could also pinpoint how much sustenance would be needed for the individual to achieve those goals. The ability to compare MEE between tasks would also be helpful. For example, a variety of lifting techniques could be evaluated to see which lifting task requires the least amount of energy, therefore, potentially identifying the most ergonomic and cost-effective approach to the lifting task.

Experimental measurement of MEE can be done via indirect calorimetry which estimates MEE based on oxygen uptake. However, a shortcoming of indirect calorimetry measurement is that it is limited to steady state tasks such as walking or cycling [1]. As an alternative, a variety of methods have been developed that estimate MEE in the literature. Some approaches involve calculating mechanical work based on body segment and center of mass motion [2, 3], consider mechanical work and locomotion efficiency [4], and predict optimal expenditure depending on the locomotion [5]. In this work, we focus on three types of MEE models: empirical predictive equations, muscle-space-based, and joint-space-based.

Empirical predictive equation formulations are typically simplistic, where whole body metabolic energy consumption is estimated by using few input variables. Some depend only on grade [6], while others are a function of a combination of speed, grade, load carriage, body mass, terrain coefficients, and oxygen consumption [7–10]. These models can be very accurate when applied and they are easy to use, but they are limited to the specific task that they are validated for like walking or running and cannot always account for tasks beyond that scope.

Muscle MEE models define MEE by utilizing the first law of thermodynamics, where MEE is a summation of mechanical work and heat liberation. Many muscle-based MEE models exist [11, 12] and some are even included as an analysis plug-in [13, 14] for the published musculoskeletal modeling software OpenSim [15], which allows researchers to easily estimate MEE based on their muscle simulation results. These MEE models rely on the Hill-Type model [16] which functions according to muscle lengths, muscle activations, and muscle stimulation. As a result, muscle-based MEE models require detailed muscle information and some estimation of muscle activity for a specific task to calculate MEE, which can sometimes be challenging to obtain.

Joint-space-based MEE models are derived from muscle MEE models but are framed in the joint-space instead of the muscle-space [17, 18]. Using a muscle-space model often requires muscle simulations that can be determined via optimization [19, 20]. The optimization uses joint moments to determine each muscle’s contribution to a body segment’s motion. Joint-space-based models eliminate the need for the muscle activation prediction and muscle information, and instead rely on commonly acquired motion data such as joint kinematics and ground reaction forces which uses motion capture in combination with force plates to calculate joint moments. The motion data allows for greater subject specificity as opposed to using empirical equation formulas which use simple measurements for height and weight but do not account for more detailed anthropometric differences between participants such as body segment lengths. Considering this, joint-space-based models require less information than is needed for muscle-space models yet provides a more subject specific MEE estimation than is provided by empirical equations.

A previous study compared seven MEE models (6 muscle-space-based and 1 joint-space-based) and found that muscle-based MEE models performed better than the joint-based model [21]. The joint-based model they used was developed by Kim and Roberts [17]. Since then, an enhanced version of that model was developed which incorporates subject-specific heat coefficients when calculating MEE instead of the more generalized coefficients used in its predecessor [18]. Roberts et al. [18] determined these heat coefficients using only level walking data and it has not been tested in other motion scenarios, such as uphill or downhill walking. Therefore, the original weighting parameter set may not be suitable for tasks beyond level walking and its performance under different circumstances has not yet been investigated. This joint-space model has the potential to estimate a larger range of tasks beyond the typical steady state scenarios previously mentioned. However, until we gain confidence in a robust parameter set that can be used for a broader range of tasks, continued testing of steady state tasks is needed before advancing to more complex, non-steady state situations. Therefore, this work is a first step towards exploring beyond steady-state walking on level ground as the Roberts model was originally validated for by testing the model with various inclines.

The foundation of this work is built on the previously validated MEE model developed by Roberts et al. [18]. In this model, the generalized heat rate coefficients incorporate weighting parameters and subject-specific parameters which include subject mass, height, age, and maximum knee torque. The coefficient weighting parameters are identified by solving an optimization problem which minimizes the root-mean-square error (RMSE) between measured and model-estimated MEE. Their work was validated for level walking at various speeds, but uphill and downhill walking was not considered. In this work, we determine how well Roberts’ model performs during level, uphill, and downhill walking at two walking speeds using published gait data. In response, new weighting parameter sets that would result in a more accurate metabolic energy consumption estimation are identified by solving a similar optimization problem through the particle swam method plus *fmincon* function in MATLAB. Finally, we compare our new results to the results found by using empirical predictive equation, muscle-spaced-based, and joint-space-based MEE models.

## 2. Method

### 2.1 Subjects

Published gait data of 12 healthy subjects are used in this study and subject information is listed in Table 1 [22]. The data set is split into a training group and a validation group. The training group contains 8 participants that are used in the weighting parameter optimization process. The validation group contains 4 participants that are used to demonstrate the success of the new parameter set found through the optimization procedure. A student’s t-test is used to ensure that the training and validation group do not have any statistical differences in mass, height, and age.

**Table 1:** Subject information for training and validation [22].

Group	Subjects	Sex	Mass (kg)	Height (m)	Age (yrs)
**Training**	1, 3, 10, 11	Male	74.72 ± 14.73	1.80 ± 0.08	25.00 ± 5.94
	2, 5, 6, 7	Female	64.30 ± 9.32	1.68 ± 0.08	22.25 ± 3.95
**Validation**	4	Male	90.58	1.72	34
	8	Male	59.25	1.70	25
	9	Female	68.72	1.70	20
	12	Female	67.28	1.69	21

### 2.2 Data collection

**Fig 1:**
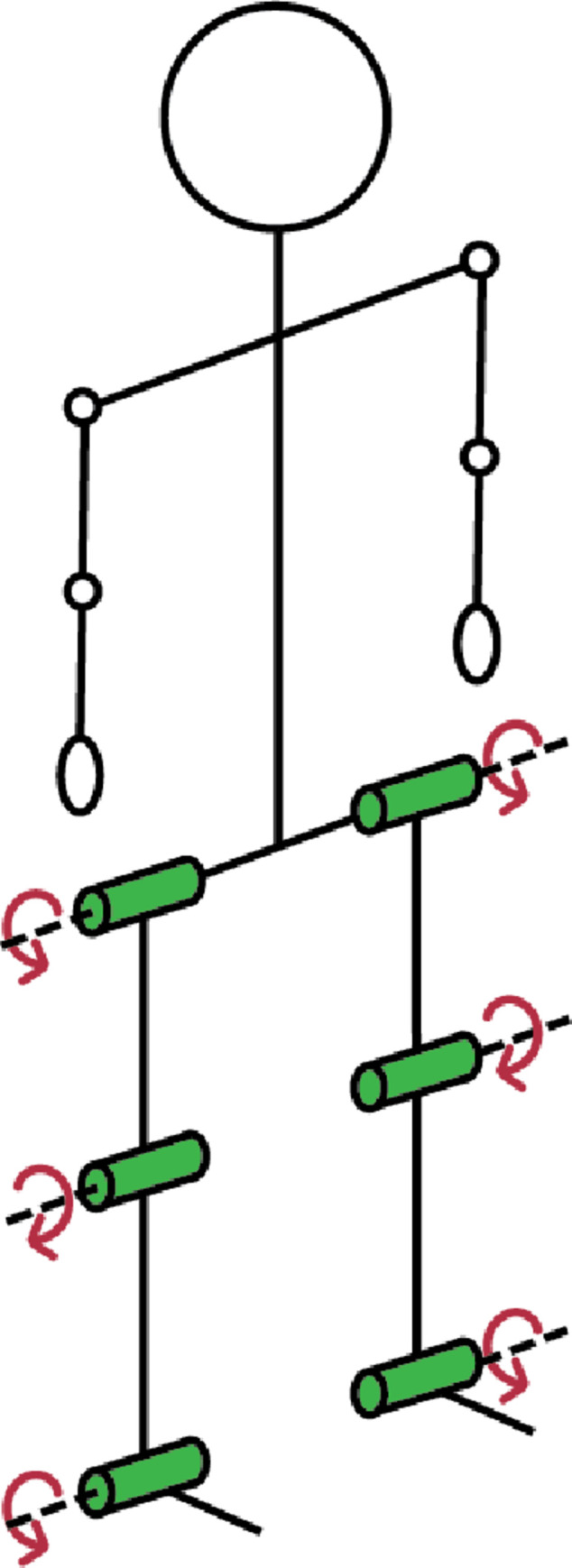
6 DOF model.

As described in previous work [21], the participants were asked to perform six walking trials with varying speed (0.8 m·s⁻ ^1^ and 1.3 m·s⁻ ^1^) and grade (-8%, 0%, and 8%) conditions. The order of trials was randomized. Pulmonary gas exchange rates were measured during all walking trials and was used to calculate measured metabolic rate (W·kg⁻ ^1^). Koelewijn et al. [21, 22] used a motion capture system and force plates to collect kinematic and kinetic data. All raw and processed experimental data were available online. The processed joint velocities, joint moments, and measured metabolic rates are used in this study. Gait cycles are averaged for each walking condition and resampled to 100 data points. The data contain 6 degrees of freedom (DOFs) which include hip flexion, knee flexion, and ankle dorsiflexion, bilaterally, as shown in Fig 1. The measured metabolic cost (J·kg⁻ ^1^·m⁻ ^1^) for each trial is calculated through dividing the metabolic rate (W·kg⁻ ^1^) by the walking speed (m·s⁻ ^1^).

### 2.3 Joint-based metabolic energy expenditure model

This study uses a metabolic energy consumption model which calculates MEE based on joint moments and joint angular velocities [18]. The intention of this paper is not to define a new MEE model, but to identify new weighting parameter sets that allow the model to handle new walking scenarios. Therefore, we will briefly describe the theory for Roberts’ model, but more details can be found in the original paper. The metabolic energy expenditure rate (W) as a function of time, ${\dot{E}}^{met}(t)$, is defined by the following equation:

$${\dot{E}}^{met}(t)={\dot{W}}^{int}(t)+{\dot{U}}^{t}(t)-{\dot{Q}}^{ext}(t)+{\dot{E}}^{o}(t)$$ (1)

${\dot{W}}^{int}$ is the work rate done by non-conservative internal forces, ${\dot{U}}^{t}$ is the internal thermal energy, ${\dot{Q}}^{ext}$ is the heat transfer rate across the system boundary, and ${\dot{E}}^{o}$ is the metabolic energy that is not related to muscles. Individually, each component can be expanded as follows. First, ${\dot{W}}^{int}$ is defined by the summation in Eq (2):

$${\dot{W}}^{int}(t)=\sum_{i=1}^{n}\tau_{i}(t){\dot{q}}_{i}(t)$$ (2)

*τ*_*i*_(*t*) is the joint torque and ${\dot{q}}_{i}(t)$ is the joint velocity at the *i*th DOF. *n* is the total number of DOF in the human model. Next, ${\dot{U}}^{t}-{\dot{Q}}^{ext}$ represents the heat exchanges occurring within the body and are defined by the summations in Eq (3):

$${\dot{U}}^{t}(t)-{\dot{Q}}^{ext}(t)=\sum_{i=1}^{n}h_{i}^{am}|\tau_{i}(t)|+\sum_{i=1}^{n}h_{i}^{sl}|\tau_{i}(t){\dot{q}}_{i}(t)|$$ (3)

$h_{i}^{am}$ is the heat coefficient associated with joint actuator torque and torque-dependent co-contraction. $h_{i}^{sl}$ is the heat coefficient associated with joint mechanical power. Lastly, ${\dot{E}}^{o}$ is the metabolic energy that is not related to muscles, i.e. basal metabolic rate. The heat coefficients, $h_{i}^{am}$ and $h_{i}^{sl}$ are formulated as follows:

$$h_{i}^{am}=(w_{0}^{am}+w_{1}^{am}M+w_{2}^{am}A+w_{3}^{am}H)(1+w_{4}^{am}\frac{\tau_{knee}^{max}}{{\tilde{\tau }}_{knee}^{max}}{\tilde{\tau }}_{i}^{max})\;(i=1,\ldots ,n)$$ (4)

$$h_{i}^{sl}=(w_{0}^{sl}+w_{1}^{sl}M+w_{2}^{sl}A+w_{3}^{sl}H)(1+w_{4}^{sl}\frac{\tau_{knee}^{max}}{{\tilde{\tau }}_{knee}^{max}}{\tilde{\tau }}_{i}^{max})\;(i=1,\ldots ,n)$$ (5)

*M*, *A*, *H*, and $\tau_{knee}^{max}$ represent the participant’s mass, age, height, and measured maximum knee torque, respectively. The published gait data did not include measured maximum knee torque for each participant, therefore we chose to estimate a maximum knee torque for men and women based on the averages found in Roberts’ original work, where men averaged 212.4 N·m and women averaged 107.6 N·m. ${\tilde{\tau }}_{knee}^{max}$ is the average human’s maximum knee torque and ${\tilde{\tau }}_{i}^{max}$ is the average human’s maximum $i^{th}$ torque. $w=[w_{0}^{am}{,w}_{1}^{am}{,w}_{2}^{am},w_{3}^{am},w_{4}^{am}{,w}_{0}^{sl}{,w}_{1}^{sl}{,w}_{2}^{sl},w_{3}^{sl},w_{4}^{sl}{]}^{T}$ represent the weighting parameters, which were solved by Roberts et al. [18] as shown in Table 2.

**Table 2:** Original weight parameter estimation for level walking from Roberts et al. [18] without normalization.

$w_{0}^{am}$	$-2.27\times 10^{-4}$
$w_{1}^{am}$	$-6.40\times 10^{-7}$
$w_{2}^{am}$	$1.61\times 10^{-6}$
$w_{3}^{am}$	$1.43\times 10^{-4}$
$w_{0}^{sl}$	$9.79\times 10^{-1}$
$w_{1}^{sl}$	$3.45\times 10^{-4}$
$w_{2}^{sl}$	$1.07\times 10^{-2}$
$w_{3}^{sl}$	$-1.46\times 10^{-1}$
$w_{4}^{am}$	$1.82\times 10^{1}$
$w_{4}^{sl}$	$-6.97\times 10^{-5}$

### 2.4 Optimization formulation

As described by Roberts et al. [18], the weighting parameters were estimated by minimizing a least squares problem. Similarly, this study calculates a new set of weighting parameters that could better estimate level, uphill, and downhill walking. The design variables are $w=[w_{0}^{am}{,w}_{1}^{am}{,w}_{2}^{am},w_{3}^{am},w_{4}^{am}{,w}_{0}^{sl}{,w}_{1}^{sl}{,w}_{2}^{sl},w_{3}^{sl},w_{4}^{sl}{]}^{T}$ for each of the walking conditions. The cost function used in our study is described in Eq (6):

$$f=\sqrt{\frac{{\left(E^{Ex}-E^{M}\right)}^{2}}{N}}$$ (6)

*E*^*Ex*^ represents the measured experimental energy expenditure and *E*^*M*^ represents the energy expenditure estimated from the model, and *N* represents the number of data points.

In response to the irreversibility of heat dissipation, it is assumed that both heat coefficients must be non-negative. Therefore, the following inequality constraints are enforced:

$$w_{0}^{am}+w_{1}^{am}M+w_{2}^{am}A+w_{3}^{am}H\geq 0$$ (7)

$$w_{0}^{sl}+w_{1}^{sl}M+w_{2}^{sl}A+w_{3}^{sl}H\geq 0$$ (8)

$$1+w_{4}^{am}\;\left(\frac{\tau_{knee}^{max}}{{\tilde{\tau }}_{knee}^{max}}\right)\;{\tilde{\tau }}_{i}^{max}\geq 0\;(i=1,\ldots ,n)$$ (9)

$$1+w_{4}^{sl}\;\left(\frac{\tau_{knee}^{max}}{{\tilde{\tau }}_{knee}^{max}}\right)\;{\tilde{\tau }}_{i}^{max}\geq 0\;(i=1,\ldots ,n)$$ (10)

Metabolic energy expenditure does not recharge via negative joint work; therefore, the following constraint is employed in Eq (11).


$$\tau_{i}(t)\dot{q_{i}}(t)+h_{i}^{am}|\tau_{i}(t)|+h_{i}^{sl}|\tau_{i}(t)\dot{q_{i}}(t)|\geq 0\;(i=1,\ldots ,n\;for\;\forall t)$$ (11)

Lastly, the total MEE is considered non-negative and bounded by the subject-specific maximum oxygen uptake (maximum *VO*_2_). Although maximum *VO*_2_ can be estimated using age and resting heart rate [23, 24], resting heart rate was not provided with the published data from Koelewijn et al. [22]. Therefore, we chose to alter this constraint by bounding the calculated total MEE by an estimated maximum MEE, *E*^*max*^, which we chose to be 110–115% of the measured MEE. The final constraint is defined in Eq (12):

$$0\leq E^{M}\leq E^{max}$$ (12)

Three optimizations were carried out via the MATLAB functions *particleswarm* and *fmincon* using a personal computer (Intel Core i7-8750H CPU @ 2.20GHz with 16.0 GB RAM) to identify three new parameter sets for level, uphill, and downhill walking, respectively. A swarm size of 100 was used and no upper or lower bounds were enforced. The particle swarm optimization would terminate when the relative change in the best objective function value was less than the default function tolerance (10^−6^). The results from the particle swarm optimization were used as the initial parameter values for the *fmincon* optimization. Default settings were used for *fmincon* optimization where function tolerance was 10^−6^ and the results from the *fmincon* optimization were used as the new parameter sets for the validation group to assess fit for the three walking conditions.

### 2.5 Statistical design and analysis

An independent-samples t-test is performed using SPSS to determine if there are any statistical differences in mass, height, and age between the training and validation groups. No outliers are identified via boxplots and both groups are normally distributed as assessed by Shapiro-Wilk’s test, *p* > 0.05. Homogeneity of variance, as assessed by Levene’s test, is present in mass (p = 0.744) and age (p = 0.791) but could not be assumed for height (p = 0.031), therefore a Welch t-test is used for height [25].

A RMSE calculation, Bland-Altman plots, and independent-samples t-tests are used to assess how well the MEE results from the validation group matched the experimental measurements for level, uphill, and downhill walking. When comparing the measured MEE to the estimated MEE for the three walking conditions using the t-test, there were no outliers identified via boxplots and all groups were normally distributed as assessed by Shapiro-Wilk’s test, *p* > 0.05. Homogeneity of variance, as assessed by Levene’s test, is present in the three walking conditions, *p* > 0.05.

In addition to calculating the RMSE for the current model, seven other MEE models are included for comparison. These included two muscle-based models [13, 14], three empirical predictive equations [7–10], and two joint-based models [17, 18]. The two muscle-based models are chosen because they are readily available within the OpenSim software which means that they are easily accessible to users. The three empirical predictive equations are included in the comparison because they have not been previously compared to both muscle-based and joint-based MEE models and they are the easiest to use. The two joint-based models are included to demonstrate the progression of the MEE model development up to the current study.

The muscle-based models and Kim and Roberts [17] were previously calculated via the published data. Therefore, the results from the Koelewijn database were directly used to calculate RMSE of the validation group for the muscle-based models and Kim’s model without manually calculating the energy consumption ourselves. Originally, Koelewijn reported net MEE calculations in the dataset, therefore we added basal metabolic rate (BMR) to the two muscle-based models and Kim’s model by using the Harris-Benedict equation [26]. The original parameter set from Roberts was used and RMSE was calculated for that group. Again, because we did not have measured maximum knee torque for each participant, we estimated those values. The empirical predictive equations were used to calculate the MEE for the validation group, and we assumed a terrain coefficient equal to 1 since the published gait data used a treadmill. More information on these models can be found in the S1 File.

## 3. Results

### 3.1 T-test for verification and validation groups

The results from the independent-samples t-test show that there is no significant statistical differences in mass (*t*(10) = -0.245; *p* = 0.811), height (*t*(7.449) = 1.142; *p* = 0.289), or age (*t*(10) = -0.417; *p* = 0.685) between the verification and validation groups.

### 3.2 Parameter sets

Three new weighting parameter sets are identified by separately solving three optimization problems dealing with level, uphill, and downhill walking. Each weighting parameter set is calculated by enforcing constraints that pertained to their respective walking condition and minimizing the objective function, using only the trials for that specific condition (8 total trials per condition). The weighting parameters, the design variables within the optimization, were not restricted to any upper or lower bounds. The *E*^*max*^ for level walking and uphill walking at both speeds is set to 110% of measured MEE. The *E*^*max*^ for downhill walking at 1.3 m·s⁻^1^ and 0.8 m·s⁻^1^ is set to 115% and 110% of measured MEE, respectively. The initial parameter set values used for the *fmincon* optimization setup and final optimal weighting parameter sets can be seen in Tables 3 and 4.

**Table 3:** Optimization initial values for weighting parameters found via particle swarm.

	Level	Uphill	Downhill
$w_{0}^{am}$	$-1.30\times 10^{3}$	$-3.27\times 10^{3}$	$-2.64\times 10^{3}$
$w_{1}^{am}$	$1.05\times 10^{2}$	$1.28\times 10^{2}$	$7.05\times 10^{1}$
$w_{2}^{am}$	$-2.76\times 10^{2}$	$-1.12\times 10^{2}$	$-1.13\times 10^{2}$
$w_{3}^{am}$	$4.16\times 10^{2}$	$-1.34\times 10^{3}$	$3.76\times 10^{2}$
$w_{0}^{sl}$	$8.70\times 10^{2}$	$4.69\times 10^{2}$	$1.06\times 10^{3}$
$w_{1}^{sl}$	$-1.24\times 10^{2}$	$-1.84\times 10^{0}$	$8.52\times 10^{1}$
$w_{2}^{sl}$	$3.16\times 10^{2}$	$-2.33\times 10^{2}$	$-2.93\times 10^{2}$
$w_{3}^{sl}$	$5.51\times 10^{1}$	$2.71\times 10^{3}$	$-4.10\times 10^{1}$
$w_{4}^{am}$	$-7.39\times 10^{-3}$	$-5.52\times 10^{-3}$	$-5.55\times 10^{-3}$
$w_{4}^{sl}$	$-7.01\times 10^{-3}$	$-5.91\times 10^{-3}$	$-7.44\times 10^{-3}$

**Table 4:** Optimal weighting parameter set values for each condition.

	Original (Roberts et al., 2016)	Level	Uphill	Downhill
$w_{0}^{am}$	$-2.27\times 10^{-4}$	$4.49\times 10^{0}$	$9.31\times 10^{0}$	$-8.94\times 10^{-1}$
$w_{1}^{am}$	$-6.40\times 10^{-7}$	$1.35\times 10^{-2}$	$-1.64\times 10^{-2}$	$-4.87\times 10^{-3}$
$w_{2}^{am}$	$1.61\times 10^{-6}$	$-4.87\times 10^{-2}$	$-1.06\times 10^{-1}$	$-8.82\times 10^{-3}$
$w_{3}^{am}$	$1.43\times 10^{-4}$	$-1.39\times 10^{0}$	$-2.10\times 10^{0}$	$8.77\times 10^{-1}$
$w_{0}^{sl}$	$9.79\times 10^{-1}$	$-1.46\times 10^{0}$	$-9.03\times 10^{0}$	$1.97\times 10^{0}$
$w_{1}^{sl}$	$3.45\times 10^{-4}$	$-1.44\times 10^{-2}$	$3.95\times 10^{-2}$	$4.37\times 10^{-4}$
$w_{2}^{sl}$	$1.07\times 10^{-2}$	$3.55\times 10^{-3}$	$2.00\times 10^{-1}$	$4.67\times 10^{-2}$
$w_{3}^{sl}$	$-1.46\times 10^{-1}$	$1.75\times 10^{0}$	$2.11\times 10^{0}$	$-9.76\times 10^{-1}$
$w_{4}^{am}$	$1.82\times 10^{1}$	$-6.64\times 10^{-4}$	$7.68\times 10^{-4}$	$3.02\times 10^{-2}$
$w_{4}^{sl}$	$-6.97\times 10^{-5}$	$2.32\times 10^{-3}$	$-2.24\times 10^{-3}$	$-1.17\times 10^{-3}$

### 3.3 RMSE calculation for all models

**Fig 2:**
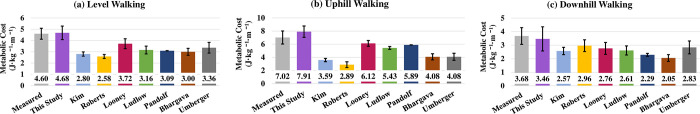
MEE model comparison and error bars represent ± 1 SD: (a) Level walking, (b) uphill walking, and (c) downhill walking.

After the parameter sets are identified for each walking case, they are used to evaluate the validation group’s energy expenditure using the joint-based MEE model. These joint-space model results are compared to the MEE estimations from other MEE models and measured MEE as shown in Fig 2. In Table 5, the validation group (4 subjects) RMSE values between the measured and estimated MEE for each model and each condition is calculated, including the RMSE for all conditions. Table 6 also includes the training group and the validation group RMSE values for each of the three walking conditions and the RMSE values for all condition.

**Table 5:** Validation group (4 participants) RMSE values (J·kg ⁻^1^·m ⁻^1^) for each model during level, uphill, and downhill walking.

Model	Level	Uphill	Downhill (N = 8)	All Conditions (N = 24)
	(N = 8)	(N = 8)		
**Kim**	1.83	3.54	1.20	2.40
**Roberts**	2.06	4.31	0.92	2.81
**Looney**	0.95	1.27	1.05	1.10
**Ludlow**	1.48	1.86	1.17	1.53
**Pandolf**	1.58	1.46	1.53	1.52
**Bhargava**	1.67	3.04	1.73	2.24
**Umberger**	1.32	3.06	1.01	2.01
**This Study**	0.45	1.56	0.82	1.05

**Table 6:** Training group (8 participants) and the validation group (4 participants) RMSE values (J·kg ⁻^1^·m ⁻^1^) during level, uphill, and downhill walking.

Group	Level	Uphill	Downhill	All Conditions
**Training**	0.41	0.68	0.46	0.53
	(N = 16)	(N = 16)	(N = 15)	(N = 47)
**Validation**	0.45	1.56	0.82	1.05
	(N = 8)	(N = 8)	(N = 8)	(N = 24)

For level walking, the average measured walking MEE is 4.60 ± 0.49 J·kg ⁻^1^·m ⁻^1^. Our study had the highest average calculation (4.68 ± 0.60 J·kg ⁻^1^·m ⁻^1^) and Roberts has the lowest average calculation (2.58 ± 0.18 J·kg ⁻^1^·m ⁻^1^). Kim (2.80 ± 0.19 J·kg ⁻^1^·m ⁻^1^), like Roberts, largely underestimated the MEE measurement. The muscle-based models, Bhargava (3.00 ± 0.31 J·kg ⁻^1^·m ⁻^1^) and Umberger (3.36 ± 0.48 J·kg ⁻^1^·m ⁻^1^), also underestimate MEE and do not fall within the range of the measured MEE. The three empirical models, Pandolf (3.09 ± 0.02 J·kg ⁻^1^·m ⁻^1^), Ludlow (3.16 ± 0.35 J·kg ⁻^1^·m ⁻^1^), and Looney (3.72 ± 0.44 J·kg ⁻^1^·m ⁻^1^), underestimate measured MEE. This study has the closest agreement and falls within the range of the measured MEE with a RMSE of 0.45 J·kg ⁻^1^·m ⁻^1^.

During uphill walking, the average measured walking MEE is 7.02 ± 0.99 J·kg ⁻^1^·m ⁻^1^. Our study has the highest estimation (7.91 ± 0.86 J·kg ⁻^1^·m ⁻^1^) and Roberts has the lowest estimation (2.89 ± 0.44 J·kg ⁻^1^·m ⁻^1^). Kim (3.59 ± 0.25 J·kg ⁻^1^·m ⁻^1^), Bhargava (4.08 ± 0.47 J·kg ⁻^1^·m ⁻^1^), Umberger (4.08 ± 0.53 J·kg ⁻^1^·m ⁻^1^), Ludlow (5.43 ± 0.22 J·kg ⁻^1^·m ⁻^1^), and Pandolf (5.89 ± 0.02 J·kg ⁻^1^·m ⁻^1^) each underestimate the measured MEE. Looney (6.12 ± 0.44 J·kg ⁻^1^·m ⁻^1^) also underestimates the measured MEE, although part of its range overlaps with the measured with a RMSE of 1.27 J·kg ⁻^1^·m ⁻^1^. Our study overestimates MEE but our range overlaps with the measured MEE with a RMSE of 1.56 J·kg ⁻^1^·m ⁻^1^.

The average measured metabolic cost during downhill walking is 3.68 ± 0.62 J·kg ⁻^1^·m ⁻^1^. Our study (3.46 ± 0.89 J·kg ⁻^1^·m ⁻^1^) has the highest estimation of MEE and Bhargava (2.05 ± 0.25 J·kg ⁻^1^·m ⁻^1^) has the lowest estimation of MEE. Kim (2.57 ± 0.27 J·kg ⁻^1^·m ⁻^1^), Pandolf (2.29 ± 0.11 J·kg ⁻^1^·m ⁻^1^), Ludlow (2.61 ± 0.34 J·kg ⁻^1^·m ⁻^1^), and Bhargava all greatly underestimate MEE. Umberger (2.83 ± 0.48 J·kg ⁻^1^·m ⁻^1^), Roberts (2.96 ± 0.43 J·kg ⁻^1^·m ⁻^1^), and Looney (2.76 ± 0.34 J·kg ⁻^1^·m ⁻^1^) also underestimate MEE, but their ranges overlap with the range of the measured MEE. Our study underestimated MEE, but our range encompasses the measured MEE with a RMSE of 1.05 J·kg ⁻^1^·m ⁻^1^.

### 3.4 Bland-Altman plots and T-test

**Fig 3:**
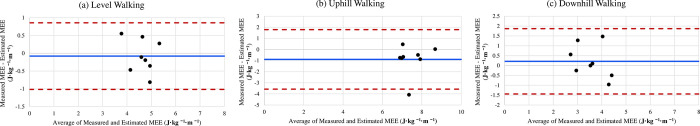
Bland-Altman Plots for Measured MEE and Estimated MEE: (a) Level walking, (b) uphill walking, and (c) downhill walking. The solid blue line represents the bias and the dashed red lines represent the 95% limits of agreement about the bias ± 1.96·SD.

The Bland-Altmann plots in Fig 3 show how closely the current study estimates MEE compared to the measured MEE. For level walking, the plot shows a bias of -0.08 J·kg ⁻^1^·m ⁻^1^ and all data points fall within the 95% limits of agreement. The plot for uphill walking shows a bias of -0.89 J·kg ⁻^1^·m ⁻^1^ and all but one data point fall within the 95% limits of agreement. Finally, the downhill walking plot shows a bias of 0.21 J·kg ⁻^1^·m ⁻^1^ and all data points fall within the 95% limits of agreement.

Our independent-samples t-test results showed that there were no statistical differences between the measured and estimated MEE values for level (*t*(14) = -0.292, *p* = 0.774), uphill (*t*(14) = -1.919, *p* = 0.076), or downhill walking (*t*(14) = 0.556, *p* = 0.587).

### 3.5 Respiratory exchange ratio values during walking trials

It is important to ensure that respiratory exchange ratio (RER) values remain in bounds of 0.7 to 1.0 when calculating metabolic rate. Koelewijn et al. [21] stated that they used the average respiratory quotient over the time for each trial where the first three minutes of walking data were discarded. We have replicated those average RER values using the raw data provided by Koelewijn’s dataset for each trial in Table 7.

**Table 7:** Average RER values (non-dimensional) for each trial from Koelewijn et al. [22]. Note that there is no data for Subject 11 during downhill walking at 1.3 m·s ⁻^1^ as it was not available in the dataset.

Subject	Level	Level	Uphill	Uphill	Downhill	Downhill
	1.3 m·s ⁻^1^	0.8 m·s ⁻^1^	1.3 m·s ⁻^1^	0.8 m·s ⁻^1^	1.3 m·s ⁻^1^	0.8 m·s ⁻^1^
**1**	0.83	0.87	0.83	0.87	0.87	0.90
**2**	0.78	0.81	0.84	0.81	0.75	0.81
**3**	0.82	0.92	0.91	0.95	0.80	0.92
**4**	0.76	0.77	0.82	0.77	0.77	0.78
**5**	0.84	0.78	0.83	0.83	0.80	0.77
**6**	0.80	0.79	0.83	0.80	0.81	0.77
**7**	0.84	0.81	0.84	0.81	0.85	0.88
**8**	0.91	0.92	0.96	0.90	0.92	0.86
**9**	0.82	0.83	0.85	0.79	0.84	0.84
**10**	0.82	0.84	0.85	0.82	0.86	0.84
**11**	0.74	0.75	0.81	0.74	N/A	0.81
**12**	0.75	0.74	0.81	0.78	0.77	0.77

## 4. Discussion

The results from the optimal weighting parameter set provided interesting results. It was anticipated that the level walking parameter set would closely align with the original parameter set because Roberts et al. [18] validated their model under a similar walking condition. Instead, the weighting values had some differences. Specifically, parameters $w_{0}^{am},w_{1}^{am},w_{3}^{sl}$ and, $w_{4}^{sl}$ increased in value from the original parameter values while the others decreased in value. The values may have altered in response to our gait data including only 6 DOF (lower extremities) instead of 42 DOF (whole body), as in Roberts’ original work. The uphill and downhill parameter sets do not have any frame of reference for comparison, but some trends can be identified among the three conditions. First, there is a consistent increase in value from downhill to level to uphill for parameters $w_{0}^{am}$ and $w_{3}^{sl}$. Additionally, there is a consistent decrease in value from downhill to level to uphill for parameters $w_{2}^{am},w_{3}^{am}$ and $w_{0}^{sl}$. The remaining parameters do not have any recognizable pattern.

The empirical predictive equations, Looney, Ludlow and Pandolf had RMSE values which ranged from 0.95 to 1.86 J·kg ⁻^1^·m ⁻^1^. All empirical predictive equations underestimated the measured MEE values and followed a consistent trend of increasing MEE estimation when adjusting from downhill to level to uphill, which is consistent with the measured MEE trend. Out of the three empirical models, Looney had the best overall performance for all three walking conditions with a RMSE values of 1.10 J·kg ⁻^1^·m ⁻^1^ for all conditions. Further, Looney had the most accurate estimation for uphill walking out of all models tested in this study. This trend aligns with what was seen in Looney’s original paper where their predictive equation had the least bias in accuracy and precision of MEE estimation when compared to Pandolf’s equation [8]. The underestimation of MEE from the empirical predictive equations was unexpected as previous publications showed close agreement with experimental measurements. This leads us to believe that it is possible that the experimental measurements from Koelewijn could be overestimations of MEE. One key advantage to using empirical predictive equations is the wide range of walking conditions which can include more extreme grades and various walking speeds. In addition, a carrying load can be taken into consideration. Despite these positive attributes, these types of models are still limited to only walking. Therefore, the shortcoming of these models would be that they are too general to use for activities beyond walking but are useful for quick calculation and estimation of energy consumption during a walking activity.

The muscle-based models, Bhargava and Umberger, consistently underestimated MEE, but Umberger had better overall performance during level and downhill walking. The current study utilized a 6 DOF model that contained information for eight major muscles bilaterally within the lower extremity but did not include musculature for the trunk or arms which may explain the underestimated MEE calculation when using the muscle-based models. An advantage of using the muscle-based MEE models is that it allows the user to break down MEE by muscle and by joint, but it appears that it may not be capable of estimating whole body MEE without muscle information throughout the body.

Surprisingly, the joint-based models, Kim and Roberts, had good agreement during downhill walking despite both being validated for level walking. They had the largest RMSE for the uphill and level walking conditions across all models tested in this work. Some reasons for this misalignment could be that only 6 DOFs were observed which was reflected in the underestimations of MEE, although Kim was originally validated with only lower extremity walking data. In addition, when we calculated MEE using Roberts’ original weighting parameter set, we used the same estimated maximum knee torque according to sex as we did for the current work. This lack of subject-specificity might explain why Roberts’ model did not perform as well as it could have. In addition, the inclusion of subject-specific parameters was meant to give Roberts’ an edge on its predecessor, but Kim’s model was more accurate during level and uphill walking.

The new weighting parameter sets from this study had the best overall performance with a RMSE value of 1.05 J·kg ⁻^1^·m ⁻^1^ for all conditions. The model overestimated MEE during level walking, but its range aligned well with the measured MEE values as shown by its RMSE of 0.45 J·kg ⁻^1^·m ⁻^1^. The Bland-Altman plot for level walking (Fig 3A) shows that the new parameter set of level walking for estimating MEE can be reasonably comparable to the measured MEE found through indirect calorimetry. The model overestimated MEE during uphill walking and had a range that deviated more than the ranges during level and downhill walking, as shown by its RMSE of 1.56 J·kg ⁻^1^·m ⁻^1^. Of all the models included in this study, the joint-space model with the new parameter set of uphill walking had the third lowest RMSE. The Bland-Altman plot for uphill walking (Fig 3B) has the largest bias among the three walking conditions and one out of the eight data points falls just outside of the limits of agreement. Despite that, the plot shows good agreement between the measured MEE and the estimated MEE values. During downhill walking, the model underestimated MEE and maintained good agreement with the measured MEE range as indicated by its RMSE of 0.82 J·kg ⁻^1^·m ⁻^1^. The Bland-Altman plot for downhill walking (Fig 3C) shows that the new parameter set of downhill walking for estimating MEE can be reasonably comparable to the measured MEE. Lastly, our t-test results support that the differences between the measured MEE and the estimated MEE values are not statistically significant for each condition.

In Roberts’ original paper, it is stated that the parameter set identified was calculated using level walking data, but that it should be capable of evaluating other tasks without altering those values. In this study, we found that the original parameter set did not perform well when using our 6 DOF lower extremity motion dataset for level walking, nor for uphill or downhill walking. Recall that the original parameter set was validated with a 42 DOF full-body data set during level walking. It is possible that the original parameters from Roberts’ paper could have performed better if a 42 DOF dataset had been evaluated. Considering this, the three new optimal parameter sets found in this study may be considered suitable for level, uphill, and downhill walking when using a 6 DOF model.

This study has the following limitations. First, the published data used in this work only included joint moments and velocities for 6 DOFs. Roberts’ model was originally validated using a 42 DOF model within Visual3D software (C-Motion, Germantown, MD, USA), capturing full body motion. While the results from our optimization is promising, it is reasonable to assume that a different parameter set could be identified when using 42 DOF data that would result in more accurate estimations of MEE. Regardless, our results align closely with the values from whole-body measurements via calorimetry. Therefore, it is reasonable to say that the parameter sets we have identified are sufficient to predict whole-body MEE while walking under the conditions described in this study if only 6 DOFs (in the lower extremities) are available. In addition, in this work we chose to estimate maximum MEE for each participant trial in response to not having access to resting heart rate from the published data, meaning that we could not estimate maximum *VO*_2_. In Roberts’ work, the maximum *VO*_2_ is the maximum possible oxygen uptake just before total exhaustion and they use it to estimate the maximum energy expenditure possible for the given task duration. It is sensible to presume that this estimated maximum MEE should be much higher than the measured MEE during a comfortable energetic task such as walking. The values we chose during optimization ranged from 10% to 15% larger than measured MEE. Although we made this estimation, the parameter sets found still produce promising results. Another limitation in this work includes the estimation of maximum knee torque for each participant. In Roberts’ work, the maximum isometric extension torque of the knee was experimentally measured for each participant. This maximum knee torque was intended to make the MEE calculation more subject-specific. Again, this measurement was not collected by Koelewijn et al. [22] in their data, therefore we estimated the value for maximum knee torque by sex. While this could be considered a crude estimation, our results demonstrate that this method is sufficient for a reasonable estimation of MEE under these circumstances. Lastly, this work has the small sample size for each walking condition for validation (n = 8). We were able to identify another open-source study that provided the necessary kinematics, kinetics, and measured metabolic expenditure, however, it would have provided us with a smaller total number of participants [27]. The data used in this study is the largest and best suited data set known to the authors.

## 5. Conclusion

In this work, we were able to identify three new optimal weighting parameter sets which can be used with 6 DOF lower extremity motion data to better estimate whole body MEE in different walking conditions. In our comparison, a variety of models perform differently under different walking conditions. The new optimal parameter sets that we found produced the lowest RMSE for level and downhill walking and the third to lowest RMSE for uphill walking. Further, there was no statistical difference between the measured MEE and the estimated MEE using the new parameter sets for the three walking conditions. The process of identifying these parameter sets via optimization leads us to believe that other parameter sets can be found for other tasks with varying availability of DOFs motion data. We believe that this work is a step towards identifying a single robust parameter set that allows for accurate estimation of MEE during any task, with the potential to mitigate a limitation of indirect calorimetry requiring lengthy steady state motion. Future work should include continued testing of Roberts’ model with a larger full-body (42 DOF) dataset as well as an expansion of tasks such as running, lifting, climbing, and jumping. In addition, the incorporation of extreme temperature and its influence on MEE could be explored and implemented in the joint-space MEE model.

## Supporting information

S1 File Metabolic energy consumption models. Describes each of the metabolic energy consumption models used within this study.
(DOCX)
