# Supplementary material for: Improved heat coefficients for joint-space metabolic energy expenditure model during level, uphill, and downhill walking
Source: PLoS One. 2022 Apr 14;17(4):e0267120. doi: 10.1371/journal.pone.0267120 (PMC9009715; doi:10.1371/journal.pone.0267120)
Supplement: S1 File — Describes each of the metabolic energy consumption models used within this study. (DOCX) [file pone.0267120.s001.docx]

**Supporting Information**

## Empirical predictive equations

Three empirical predictive equations were included in our energy consumption comparison. We used the following three formulations to calculate the energy consumption of our validation group. The load carriage decision aid (LCDA) is an equation that is capable of estimating energy expenditure while walking on level or graded terrain (Looney et al., 2019). It was developed to specifically target healthy, military age personnel. The walking equation is defined as:

$$EE=1.44+1.94S^{0.43}+0.24S^{4}+0.34SG(1-{1.05}^{1-{1.1}^{G+32}})$$

$EE$ represents gross energy expenditure (W·kg ⁻^1^), $S$ represents the walking speed (m·s ⁻^1^), and $G$ represents the terrain grade (%).

A previous energy consumption equation was developed by Pandolf et al. (1977) which estimates energy consumption during level and uphill walking. It is defined as:

$$EE_{level/uphill}=1.5M+2\left( M+L \right)\left( \frac{L}{M} \right)^{2}+\mu(M+L)(1.5S^{2}+0.35SG)$$

$EE_{level/uphill}$ represents the gross energy expenditure (W), $M$ represents the nude weight of the person (kg), $L$ represents the load the person is carrying (kg), $S$ represents the walking speed (m·s ⁻^1^), $G$ represents the slope grade (%), and $\mu$ represents the terrain coefficient. Santee et al. (2003) expanded this equation so that it could accommodate downhill walking by subtracting a correction factor from $EE_{level/uphill}$. The correction factor, $CF$, is defined as:

$$CF= \mu\left( \frac{SG\left( M+L \right)}{3.5}-\frac{\left( M+L \right)\left( G+6 \right)^{2}}{M}+\left( 25-S^{2} \right) \right)$$

The final equation for downhill walking is defined as:

$${EE}_{downhill}={EE}_{level/uphill}-CF$$

The third empirical model used in this study is known as the Minimum Mechanics model (Ludlow and Weyand, 2017). It can calculate energy expenditure using walking speed and grade. It is defined as follows for level and inclined walking:

$$\dot{V}_{{O_{2-gross}}_{level/uphill}}=\dot{V}_{O_{2-rest}}+0.32G+3.28+2.66S^{2}\left( 1+0.19G \right)$$

$\dot{V}_{O_{2-gross}}$ represents is the body’s gross metabolic rate (ml $O_{2}$∙kg ⁻^1^∙min ⁻^1^), $\dot{V}_{O_{2-rest}}$ represents the supine resting metabolic rate (BMR), $G$ represents the positive inclined grade (%), and $S$ represents the walking speed (m·s ⁻^1^). When calculating energy consumption while walking downhill the energy expenditure was calculated as follows:

$$\dot{V}_{O_{2-gross_{downhill}}}=\dot{V}_{O_{2-rest}}+0.73(3.28+2.66S^{2})$$

## Muscle-Based Models

In Koelewijn et al. (2019), two muscle models, Bhargava et al. (2003) and Umberger et al. (2002), were included in their comparison study. We extracted the processed results (MEE) for those models via Koelewijn’s open-source database and used the values associated with our validation group. Although we did not manually calculate the energy consumption for these two models, we used the same participants, participant inputs, and compared the results to the same experimentally measured energy consumption. Complete detail on these models can be found in their respective papers and are summarized in Koelewijn’s Appendix. The two models will briefly be described.

Bhargava et al. (2013) defined the rate of energy consumption $\dot{E}$ (W), as:

$$\dot{E}=\dot{A}+\dot{M}+\dot{S}+\dot{B}+\dot{W}$$

$\dot{A}$ represents the activation heat rate, $\dot{M}$ is the maintenance heat rate, $\dot{S}$ is the shortening heat rate, $\dot{B}$ is the basal metabolic rate, and $\dot{W}$ is the work rate.

Umberger et al. (2002) defined the rate of energy expenditure per kilogram of muscle tissue, $\dot{E}$ (W·kg⁻^1^), as:

$$\dot{E}=\dot{h_{A}}+\dot{h_{M}}+\dot{h_{SL}}+\dot{w_{CE}}$$

$\dot{h_{A}}$ represents activation heat rate, $\dot{h_{M}}$ represents the maintenance heat rate, $\dot{h_{SL}}$ represents the shortening/lengthening heat rate, and $\dot{w_{CE}}$ represents the mechanical work rate of the contractile element.

## Joint-Based Model

The model developed by Kim and Roberts (2015) was the predecessor to Roberts et al. (2016). The final results of Kim’s model were made available in Koelewijn’s published database, as were the muscle-based models. Therefore, we did not manually calculate the energy consumption for our validation group, but simply extracted the final values from the database. More complete detail on Kim’s model can be found in Koelewijn’s comparison paper. The overall equation for metabolic rate for Kim’s model is defined as:

$$\dot{E_{i}}=\dot{h_{i}}+p_{i}$$

$\dot{E_{i}}$ is the metabolic rate (W) at joint $i$, $\dot{h_{i}}$ is the heat rate, and $p_{i}$ is the joint power.
